# Protective factors enhancing resilience in children of parents with a mental illness: a systematic review

**DOI:** 10.3389/fpsyg.2023.1243784

**Published:** 2023-12-15

**Authors:** Marieke Van Schoors, Elke Van Lierde, Kim Steeman, Lesley L. Verhofstadt, Gilbert M. D. Lemmens

**Affiliations:** ^1^Familieplatform VZW, Antwerp, Belgium; ^2^Department of Experimental Clinical and Health Psychology, Ghent University, Ghent, Belgium; ^3^Department of Head and Skin – Psychiatry, Ghent University, Ghent, Belgium; ^4^Department of Psychiatry, Ghent University Hospital, Ghent, Belgium

**Keywords:** mental illness, parents, COPMI, protective factors, review

## Abstract

**Objectives:**

A systematic review was conducted to (1) investigate protective factors enhancing resilience in children of parents with a mental illness (COPMI), and (2) examine theoretical and methodological issues in the existing literature.

**Method:**

Following guidelines for systematic reviews, searches were performed using Web of Science, Pubmed and Embase. After screening 5,073 articles 37 fulfilled inclusion criteria and were extracted for review. Results of the present review indicate that there are several ways to help build resilience in COPMI. More specifically, five protective factors emerged from the reviewed literature: Information, Support, Family functioning and Connectedness, Child coping, and Parenting.

**Discussion:**

Research on protective factors in children confronted with parental mental illness is still scarce and for some factors no clear conclusions can be drawn based on the available evidence. To further our understanding of the building blocks and underlying mechanisms of resilience in COPMI, additional rigorously designed studies are needed.

## Introduction

One in every eight people in the world lives with a mental illness (WHO, 2022). Having a mental illness and its symptoms may impact a person’s life in several ways. It reduces general health and quality of life (Evans et al., 2007), affects social relationships (Leufstadius et al., 2008), and lowers the person’s opportunities for employment (Mechanic et al., 2002). In addition, suffering from a mental illness impacts a person’s family (life) and (when applicable) parenting. Indeed, a mental illness may undermine parents’ capacity to parent and impact the quality of parent–child interactions (Goodman and Gotlib, 1999; Goodman, 2007). For example, parents with depression express fewer positive emotions, have less child-oriented and more parent-oriented concerns (Dix et al., 2004), and exhibit behavior towards their children that is more hostile and less positive and engaging compared to parents without depression [e.g., (Lovejoy et al., 2000)].

Approximately 17–25% of all children worldwide live with at least one parent who has a mental illness (Maybery et al., 2009; van Santvoort et al., 2014). There is abundant empirical evidence for a profound impact on these children (e.g., Leijdesdorff et al., 2017), both at the level of their individual functioning and interpersonal functioning. Children of parents with a mental illness (COPMI) are in particular at risk for developing cognitive, emotional or behavioral difficulties themselves (Göpfert, 1996; Gladstone et al., 2014) or having mental health problems as a child and/or in later life (Leijdesdorff et al., 2017; Brummelhuis et al., 2022). Although results vary between studies, a meta-analysis indicated that up to 55% of the offspring of parents with a serious mental illness (e.g., schizophrenia, major depression, bipolar disorder or severe borderline personality disorder) develop a mental illness themselves (e.g., depression, anxiety, disruptive disorder) and are therefore 2.5 times more at risk compared to children of healthy parents (Rasic et al., 2014).

Despite the challenges, some COPMI, however, show resilience and manage to grow up without significant difficulties (Collishaw et al., 2016). Resilience can be defined as healthy or adaptive functioning over the passage of time in the aftermath of adversity (Southwick et al., 2014). In other words, in the presence of adversity, protective factors supersede the risk of developing mental problems or other difficulties, and provide a more positive outcome than might be expected in the context of such adversity (Windle, 2011; Fletcher and Sarkar, 2013). Importantly, resilience cannot be conceptualized as just the opposite of risk (Stainton et al., 2019). Thus, notwithstanding the large amount of evidence on vulnerability and risk in COPMI [as summarized in different reviews; e.g., (Leijdesdorff et al., 2017; Lawrence et al., 2019; Ayano et al., 2021)], results from research with a unique focus on protective factors and resilience in COPMI are also needed. While a growing number of researchers have investigated different protective factors, such as social support (Collishaw et al., 2016) or providing adequate information for children (Bartsch et al., 2014), their results have not yet been summarized. Moreover, this review also aims to extend existing knowledge on the effectiveness of preventive programs or interventions in reducing vulnerability in COPMI [as summarized in the review of Havinga et al., 2021]. Although these programs or interventions have been shown to be effective (Havinga et al., 2021), not all children have access to these interventions nor are they motivated to attend. As the current review aims to enhance resilience in all COPMI, both protective factors in interventions *and* in the daily lives of COPMI and their family are evaluated. A better understanding of all protective factors is needed to best support COMPI and to optimize care provided by the children’s informal and professional network.

In summary, the primary aim of this paper is therefore to provide a summary and commentary on the current evidence regarding protective factors enhancing resilience in COPMI. The secondary aim is to examine theoretical and methodological issues in the existing literature and formulate recommendations for future research.

## Method section

The review followed a strict scientific methodology in accordance with the PRISMA guidelines (Page et al., 2021) to ensure comprehensiveness, minimal bias and reliability. Therefore, the following steps were taken: (1) formulation of the scope of the review and research question, (2) thorough literature search in several databases, (3) detailed data-extraction, and (4) integration of the major findings. A systematic review was chosen above a meta-analysis, as we wished to integrate quantitative and qualitative findings in order to ensure a comprehensive overview.

### Literature search and inclusion criteria

Pubmed, Web of Science and Embase were searched using the following keywords: [Protective factor OR Resilienc* OR Emotional adjustment OR Posttraumatic growth] AND [Child* OR Adolescen* OR Adult Child* OR Offspring] AND [Parent* OR Mother* OR Maternal* OR Father* OR Paternal* OR Intergenerational* OR Parenting OR Parent–child relation*] AND [Anxiet* OR Anxious OR Bipolar* OR Dissociative* OR Eating disorder* OR Mood disorder* OR depress* OR affective disorder* OR Neurotic disorder* OR Personality disorder* OR Schizophren* OR psychot* OR psychos* OR Mentally Ill* OR psychopatholog* OR Mental health OR Mental illness*].

Studies selected for inclusion examined protective factors enhancing resilience in children of parents with a mental illness. The following studies were excluded (1) studies exclusively focusing on parental psychiatric “symptoms” (e.g., cohort study examining parental depressive symptoms), (b) studies examining factors which are not malleable (e.g., intelligence, SES), (c) studies published in languages other than English, (d) non-empirical articles (e.g., case reports, reviews, commentaries, book reviews, conference abstracts and dissertations), and (e) studies exclusively focussing on the perinatal period (children aged 0–2 years) or adult COPMI experiences. Reference lists of the selected papers were reviewed to ensure inclusion of all relevant papers.

### Study selection

The database search was undertaken in June 2022, identifying 5,073 unique papers. The first and second authors independently screened all titles for decisions regarding exclusion, with 93% agreement. Disagreements were discussed and resolved. The 374 remaining abstracts were then screened for exclusion, again by the first and second author, with an agreement rate of 81%. Disagreements were discussed and resolved. Finally, the first (100%) and second author (20%) screened the full texts of the remaining 88 studies for final decisions regarding inclusion, with 93% agreement. Disagreements were discussed and, if necessary, a third reviewer was consulted. Finally, 26 studies were selected. Eleven articles were added based on reference chaining, resulting in a final set of 37 papers (see Supplementary Figure S1).

### Data extraction

Data from the included studies was extracted in a systematic and standardized way, using a data abstraction sheet (available upon request). On this data abstraction sheet, the following study aspects were identified: (a) study characteristics such as year of publication and first author; (b) which, if any, theoretical framework was used; (c) methodological aspects, such as design, sample size and unit of measurement, and (d) a summary of the general findings. Full texts to which we had no access, were requested from the first author.

### Scientific evaluation of the included studies

Each included study was rated with respect to its scientific merit using the Mixed Method Appraisal Tool (MMAT; Hong et al., 2018). Quantitative studies were evaluated for appropriateness of sampling strategy, representativeness of the sample, measurement reliability and appropriateness of available data and statistical analysis. Qualitative studies were evaluated for their scientific purpose, appropriateness of design and analysis, grounding results in examples and coherence between data sources, collection, analysis and interpretation. Each aspect was rated on a “yes,” “no,” “cannot tell” scale. In line with the recommendations of Hong et al. (2018), an overall score per study was not calculated. Instead, we provided a detailed representation of the ratings of each criterion to better inform about the quality of the included studies; see Supplementary Table S1. No studies were excluded based on their scientific evaluation.

## Results

### PART 1: characteristics of reviewed studies

The methods and findings of the 37 reviewed studies are summarized in Supplementary Table S2. Most of the reviewed studies used quantitative methods (*n* = 32; 86%), four studies used qualitative methods (11%) and in the remaining study a mixed method design was used (3%). More than half of the studies were cross-sectional (*n* = 22; 59%); the remaining studies were longitudinal (*n* = 15; 41%). In the included studies, responses of parents (*n*_studies_ = 34), children (*n*_studies_ = 35), teachers (*n*_studies_ = 2) and/or clinicians (*n*_studies_ = 3) were collected and analysed. Sample sizes ranged from zero to 331 children and zero to 331 parents. In 17 studies, *only* mothers were included; overall there were 83% maternal responses. A wide variety of parental mental illnesses were included, with mood and anxiety disorders as the most frequently represented (see Supplementary Table S2 for an overview of the included parental mental illnesses).

### PART 2: narrative summary of reviewed studies

Five protective factors emerged from the reviewed literature: (1) Information, (2) Support, (3) Family functioning and Connectedness, (4) Child coping, and (5) Parenting. Within each of the following subsections, a brief explanation of the protective factor is given, followed by the number and type of included studies and a narrative summary of the findings across studies. Where applicable, qualitative results are presented first. For more details, see Supplementary Table S2.

#### Information

Information refers to the provision of information and psycho-education to children in order to increase their knowledge about the mental illness, the treatment and its consequences for themselves and the family life. This construct was addressed in two qualitative studies (Griffiths et al., 2012; Bartsch et al., 2014) and one mixed method study (Maybery et al., 2005). Across studies, the importance of being informed was emphasized. Children needed information about the diagnosis and its symptoms (Bartsch et al., 2014). Indeed, results indicated that COPMI benefit from education about their parent’s difficulties (Maybery et al., 2005; Griffiths et al., 2012), by the parent themselves or by a mental health care professional (Maybery et al., 2005).

#### Support

Support refers to assistance, encouragement, and care as perceived by the child, both from the informal and professional network. Support was addressed in four qualitative studies (Dunn, 1993; Griffiths et al., 2012; Bartsch et al., 2014; Kadish, 2015), 10 quantitative studies (Garber and Little, 1999; Boyd and Waanders, 2013; Chen, 2013; van Loon et al., 2014, 2015; Collishaw et al., 2016; Charrois et al., 2017; Iacono et al., 2018; Vakrat et al., 2018; Radicke et al., 2021) and one mixed method study (Maybery et al., 2005). Across the included studies, emotional support was addressed in particular *or* the type of support (e.g., emotional vs. practical vs. financial support) was not specified. In the qualitative and mixed method studies, support was considered as an important protective factor in helping the children get through difficult moments. Different sources of support were described: a second, healthy parent (Bartsch et al., 2014; Kadish, 2015), siblings (Maybery et al., 2005; Bartsch et al., 2014; Kadish, 2015), extended family members (e.g., grandparent/aunt; Dunn, 1993; Griffiths et al., 2012; Bartsch et al., 2014), friends or neighbours (Dunn, 1993; Maybery et al., 2005) or a stable role model in the community (e.g., teacher/coach; Dunn, 1993; Bartsch et al., 2014). Sources of support that were available on a regular basis made a substantial difference in the children’s life (Dunn, 1993) by providing a welcoming and supportive place when the parent was unwell (Dunn, 1993; Maybery et al., 2005; Kadish, 2015). When needed, children benefit from support of people with expertise (i.e., health care provider or friend with shared lived experiences; Maybery et al., 2005; Griffiths et al., 2012).

Ten quantitative studies investigated the association between perceived support and the adaptation of COPMI. First, more *overall support* (e.g., from friends, family) was related to better health-related quality of life (Radicke et al., 2021) and higher competence (i.e., better functioning and no psychopathology; Garber and Little, 1999). Second, more support from the *healthy parent* was related to fewer mood and behavior disorder symptoms (Collishaw et al., 2016), fewer depressive symptoms (Mahedy et al., 2018), and a decreased likelihood of having a psychiatric disorder; both in cross-sectional (Vakrat et al., 2018) and longitudinal (Collishaw et al., 2016; Mahedy et al., 2018) studies. Third, support from the *extended network* was examined. More support from extended family members was associated with less depressive symptoms in one study (Boyd and Waanders, 2013). In another study, however, the association between family support and child problems (i.e., internalizing and externalizing problems) was not significant in cross-sectional nor longitudinal analyses (van Loon et al., 2015). More teachers’ support was related to higher educational aspirations (Chen, 2013). Moreover, support from within high-quality child-care was reported to buffer the impact of parental mental illness on child hyperactivity and inattention (Charrois et al., 2017). Finally, three studies addressed support provided by the *parent with a mental illness*. Van Loon et al. (2015) did not find a significant association between parental support and children internalizing and externalizing problems. Notably, parents with a mental illness showed lower parental support compared to healthy parents (van Loon et al., 2014; Iacono et al., 2018). Iacono et al. (2018) conducted mediation analyses, showing that having a parent with a mental illness was associated with elevated externalizing symptoms in children via insufficient parental support. In contrast, such an indirect effect was not found by the path analyses of van Loon et al. (2014).

#### Family functioning and connectedness

Family functioning refers to the ways in which the family as a whole operates, and contains several family functioning domains, e.g., connectedness, communication, roles. As one family domain, namely connectedness, was especially mentioned in the included literature, this will be described separately. Connectedness refers to the feeling of belonging to, or having affinity with, particular people.

##### Family functioning

Nine quantitative studies (Black et al., 2003; Foster et al., 2008; Riley et al., 2009; van Loon et al., 2014; Freed et al., 2015; van Loon et al., 2015; Havinga et al., 2017; Iacono et al., 2018; Radicke et al., 2021) addressed the association between general family functioning and child outcomes, however, results were mixed. In two studies, family functioning emerged as a protective factor. According to Havinga et al. (2017) balanced family functioning (i.e., average levels of family cohesion and family adaptability) was associated with a decreased likelihood of mood/anxiety disorders (Havinga et al., 2017). Likewise, Foster et al. (2008) found that better family functioning (i.e., more cohesion and expressiveness, less conflict) was associated with less internalizing problems (Foster et al., 2008). Five other studies in which family functioning was examined did not provide support for family functioning as a protective factor. More specifically, general family functioning was not significantly associated with health-related quality of life in children and adolescents (Radicke et al., 2021), nor with children’s emotional and behavioral problems or adaptive skills (Riley et al., 2009) and with internalizing and externalizing problems at baseline and 2 years later (van Loon et al., 2015). Studies focussing on specific family functioning domains indicated that family expressiveness was not associated with child internalizing, externalizing problems and child psychopathology (Freed et al., 2015). Moreover, Black et al. (2003) did not find a significant association between a psychiatric diagnosis in COPMI and several specific domains of family functioning measured 2 years earlier (i.e., family problem solving, family communication, family roles, affective involvement within the family) (Black et al., 2003). Finally, parents with a mental illness reported lower family structure (i.e., organization and consistency in the family) and control (Iacono et al., 2018), as well as less expressiveness and more family conflict (van Loon et al., 2014) than parents without mental illness. Moreover, Iacono et al. (2018) conducted mediation analyses, showing that having a parent with a mental illness was associated with elevated internalizing, externalizing and long term depressive symptoms via family structure, and with externalizing symptoms, long term depressive symptoms and substance use via family control. Path analyses of van Loon et al. (2014) showed that having a parent with a mental illness was directly related to having more internalizing problems in adolescence, but no longer to more externalizing problems after inclusion of the family factors. Indeed, families with a mentally ill parent showed more family conflict, *which in turn* was associated with having more externalizing problems in adolescence (van Loon et al., 2014).

##### Connectedness within the family

Eight studies addressed the association between family connectedness and child adaptation (Garber and Little, 1999; Schiffman et al., 2002; Black et al., 2003; Lewandowski et al., 2014; van Loon et al., 2014; Freed et al., 2015; Keeton et al., 2015; Mahedy et al., 2018). In six of them, evidence was found for family connectedness as a protective factor. More connectedness within the family system as a whole was related to better competence (i.e., better functioning and no psychopathology; Garber and Little, 1999), lower internalizing symptoms in offspring younger than 13 years old (Freed et al., 2015) and a decreased likelihood of a psychiatric diagnosis (Black et al., 2003). In addition, the connectedness within specific family subsystems proved to be important. More closeness within the couple relationship was associated with more paternal emotional support which *in turn* was associated with less adolescent depressive symptoms (Mahedy et al., 2018). More closeness between children and both their parents was related to lower rates of lifetime schizophrenia (Schiffman et al., 2002). Finally, more closeness between siblings protected against the negative outcomes associated with parental psychological distress: while parent psychological distress was associated with child psychological symptoms in children reporting a poor quality sibling relationship, this was not the case in children reporting a good quality sibling relationship (Keeton et al., 2015). Finally, in two studies, however, no association was found between family connectedness and internalizing and externalizing symptoms (van Loon et al., 2014), or absence of a lifetime psychiatric diagnosis (Lewandowski et al., 2014).

##### Connectedness with friends

One qualitative (Bartsch et al., 2014) and three quantitative studies (Boyd and Waanders, 2013; Chen, 2013; Collishaw et al., 2016) addressed the children’s connectedness with friends. Children coped better when they had good social skills (Bartsch et al., 2014). In addition, more prosocial friendships were related to less conduct disorder symptoms (Chen, 2013); whereas better child social skills were associated with lower depressive symptoms, especially in case of low levels of positive parenting skills (Boyd and Waanders, 2013). Finally, better peer relationship quality (child and parent report) was related to sustained mental health during the study period (throughout 4 years) and fewer mood and behavior disorder symptoms at final assessment (Collishaw et al., 2016).

#### Coping

Coping refers to the thoughts and behaviors that are used by the child to manage their stressful situation. One qualitative (Kadish, 2015) and eleven quantitative (Garber and Little, 1999; Langrock et al., 2002; Jaser et al., 2007, 2008, 2011; Fear et al., 2009; Compas et al., 2010; van Loon et al., 2015; Monti and Rudolph, 2017; Thompson et al., 2017; Gruhn et al., 2019) studies reported on child coping styles. According to the study of Kadish (2015), children used different adaptive coping behaviors (e.g., early self-care, caring for the ill parent and/or siblings, actively trying to be different and more productive than the parent) and therefore believed that they possessed the necessary resilience to survive their childhood challenges. Moreover, high-competence children (i.e., children who continued to function well throughout 2 years of study) were found to use more positive coping compared with decreased-competence children (Garber and Little, 1999), whereas the use of confrontation as an active coping style predicted fewer internalizing problems 2 years later (van Loon et al., 2015). In contrast, active coping (i.e., confrontation and seeking social support) was not associated with internalizing and externalizing problems at baseline (van Loon et al., 2015).

The other included studies on coping focused on two types of adaptive coping, that is (1) primary control coping (i.e., children using problem solving, emotional expression and emotional modulation) and (2) secondary control coping (i.e., children using acceptance, positive thinking, distraction and cognitive restructuring).

##### Primary control coping

The use of primary control strategies was investigated in four studies. In two studies, primary control coping emerged as a protective factor with higher use of primary control strategies being associated with higher levels of observed positive mood (Jaser et al., 2011), less affective problems (Jaser et al., 2011), and less aggression (Fear et al., 2009). However, no significant association was found with observed sadness (Jaser et al., 2011) or with child symptoms (anxiety/depression and aggression; Langrock et al., 2002). Finally, the type of stressor seemed to matter: according to Jaser et al. (2007), greater use of primary control coping with *peer stress* predicted fewer child-reported symptoms (anxiety/depression and aggression), whereas greater use of primary control coping with *family stress* predicted more child-reported symptoms of anxiety and depression (Jaser et al., 2007).

##### Secondary control coping

Seven studies addressed the association between secondary control coping and child outcomes. Higher use of secondary control coping was related to less anxiety and depressive symptoms (Langrock et al., 2002; Jaser et al., 2007, 2008; Fear et al., 2009), less affective problems (Jaser et al., 2008, 2011), and less aggression/oppositional defiant problems (Jaser et al., 2008; Fear et al., 2009). The latter association, however, was not found to be significant in the study of Langrock et al. (2002). Associations between secondary control coping and observed emotions during mother–child interactions were examined in two studies. Higher levels of this child coping style was related to higher levels of observed positive mood (Jaser et al., 2011; Gruhn et al., 2019) and lower levels of observed hostility (Gruhn et al., 2019) in COPMI. To the contrary, no significant association was found for observed anxiety (Gruhn et al., 2019) and observed sadness (Jaser et al., 2011; Gruhn et al., 2019). Furthermore, increases in the use of secondary control coping were observed following an intervention, which *in turn* were associated with reduced anxious-depressive symptoms, internalizing symptoms and externalizing symptoms (Compas et al., 2010). Finally, Jaser et al. (2008) and Langrock et al. (2002) conducted mediation analyses, showing that secondary control coping mediated the association between observed maternal sadness and child symptoms (depressive symptoms, affective problems and oppositional defiant problems), and between parental withdrawal and child symptoms (anxiety and depressive symptoms), respectively.

##### Primary control coping and secondary control coping combined

In two studies, primary and secondary control coping were combined in a composite score. Higher use of both adaptive coping strategies was significantly associated with less depressive symptoms (Monti and Rudolph, 2017; Thompson et al., 2017). In addition, the use of these adaptive coping strategies was related to lower youth depression (at baseline and 4 years follow-up) in girls irrespective of maternal depression; in boys exposed to maternal depression a decrease in youth depression over time was observed (Monti and Rudolph, 2017). Thompson et al. (2017) also found that daughters’ adaptive coping mediated the association between maternal depressive symptoms and daughters’ depressive symptoms (Thompson et al., 2017).

#### Parenting

In one qualitative (Bartsch et al., 2014) and 13 quantitative studies (Feng et al., 2008; Foster et al., 2008; Garai et al., 2009; Riley et al., 2009; Compas et al., 2010; Boyd and Waanders, 2013; Chen, 2013; Lewandowski et al., 2014; Sellers et al., 2014; van Loon et al., 2014, 2015; Collishaw et al., 2016; Loechner et al., 2020), the role of parenting was addressed. According to Bartsch et al. (2014), acknowledging and validating the children’s emotions and responses to the illness was a protective parental characteristic for COPMI. In the quantitative studies, the following parenting concepts were addressed: positive parenting, parental warmth and parental monitoring. First, eight studies focused on *positive parenting*. Positive parenting can be described as warm child-centred behavior, with positive reinforcement, listener responsiveness and quality time between the ill parent and the child. More positive parenting was related to less internalizing symptoms (Foster et al., 2008), less externalizing symptoms (Garai et al., 2009), less depressive symptoms (Boyd and Waanders, 2013), less emotional and behavioral problems and more child adaptive skills (Riley et al., 2009). Positive parenting partially mediated the association between maternal depression and children’s emotional and behavioral problems, and fully mediated the association between maternal depression and children’s adaptive skills (Riley et al., 2009). Furthermore, increases in positive parenting were observed following an intervention (i.e., the Family Group Cognitive-Behavioural Preventive Intervention), which *in turn* was associated with reduced externalizing and depressive symptoms (Compas et al., 2010). However, also non-significant associations between positive parenting and child outcomes were found, that is with positive mood and active emotion regulation (Feng et al., 2008), depressive symptoms (Loechner et al., 2020), externalizing problems (Foster et al., 2008), internalizing problems (Garai et al., 2009), and the absence of lifetime psychiatric diagnosis (Lewandowski et al., 2014). Second, two studies addressed *parental* warmth (i.e., an accepting, caring, and supportive parenting style), with more parental warmth being related to fewer behavior disorder symptoms 4 years later (Collishaw et al., 2016) and to less disruptive symptoms, but not to child depressive symptoms (Sellers et al., 2014). Third, three studies focused on *parental monitoring*. Parental monitoring reflected the parent’s knowledge of the child’s whereabouts and activities. Higher parental monitoring was related to less conduct disorder symptoms, higher educational aspirations (Chen, 2013), less externalizing symptoms (van Loon et al., 2015) and less internalizing problems over time (van Loon et al., 2015). Moreover, parents with a mental illness showed less monitoring of the child than parents without mental illness, which *in turn* was associated with more adolescents externalizing problems [Path analyses; (van Loon et al., 2014)].

### Part 3: evaluation of the literature

#### Theoretical consideration

In the majority of the studies (*n* = 27; 73%), no theoretical framework was specified as guiding the research questions or selection of the variables. Failure to use theoretical frameworks risks limiting progression of the field as advances cannot be made if theories go untested and unrevised.

#### Methodological consideration

The heterogeneity across and within studies with regard to sample characteristics and operationalisations of used constructs and outcomes make it difficult to generalize. For example, half of the studies focused on parents with depression (*n* = 24; 65%), however, other – sometimes described as more severe – psychiatric diagnoses (e.g., psychosis or personality disorders) were also included in other studies. In addition, the treatment status of the parent (being on or off treatment) was seldom reported. Finally, all studies relied on self-reports despite known drawbacks associated with this method, e.g., social desirability (Schwartz et al., 1998). Different research methods (e.g., observations, questionnaires, interviews) combined with different units of measurement (e.g., parents, child, clinicians, teachers) are indispensable in furthering our understanding of child resilience in response to a parent suffering from a mental illness.

## Discussion

The current systematic review summarizes empirical evidence about protective factors for children growing up with a parent suffering from a mental illness. Although it has been acknowledged that some COPMI adapt better than others (Collishaw et al., 2016), little is known about the contributing factors and underlying mechanisms. Based on the present review, in which results from 37 studies were included, evidence for five protective factors was found. A first protective factor is the availability of clear and understandable *information*, as COPMI benefit from gaining a better understanding of their parents’ mental illness and its associated difficulties. Second, *support* from different sources (e.g., co-parent, siblings, family members, confidant) is found to play a substantial role. A third protective factor that arose from the reviewed literature was *family functioning and connectedness* within the family as a whole and with specific family members or friends. Although better family functioning and higher connectedness are found to buffer against poor outcomes in some studies, the available evidence on these constructs remains inconclusive. The way in which COPMI tend to *cope* with stressors is the fourth factor that is found to affect child outcomes. The use of adaptive coping strategies, such as acceptance, problem solving or positive thinking, can foster resilience in COPMI. Finally, the role of *parenting* was addressed in this review, showing that parental warmth and monitoring are protective elements in the lives of COPMI. These children may also benefit from positive parenting, but to date, results for this parenting style are less convincing.

Results of the present study indicate that there are several ways to help build resilience in COPMI. Nevertheless, research on protective factors in families confronted with parental mental illness is still scarce and for some factors no clear conclusions can be drawn based on the available evidence, warranting further research. In addition, the conclusions of this review are hampered by some limitations of the review process on the one hand and limitations of the included studies on the other hand. Regarding the current review, a first limitation is that the included studies are – in line with the scope of the review – limited to those focussing on *protective factors* for COPMI. However, resilience in COPMI is most likely determined by a combination of several complex factors, both in parent and child (e.g., protective factors, risk factors, social demographic variables, personality characteristics of COPMI, …). To best predict resilience in COPMI, all influencing factors should be taken into account. Second, only three databases (i.e., Web of Science, Pubmed, and Embase) are searched and only English articles are included. As a consequence, possibly not all relevant results on protective factors for COPMI are included in this review. Finally, results which are not statistically significant can still have clinical relevance. However, due to publication bias, it is more likely that non-significant results are not included in this review, hampering again nuanced conclusions.

Also limitations of the included studies should be taken into account when considering the conclusions of this review. First, the included studies focused on a limited amount of protective factors and outcome variables, i.e., in most studies only one to three factors are taken into account. This way complex interactions between co-occurring elements in the lives of COPMI are overlooked. In addition, as most protective factors impact specific outcomes (Chen, 2013) and the likelihood of sustained good mental health in offspring increases with the total number of protective factors present (Collishaw et al., 2016), studies should include more protective factors in order to evaluate their unique contribution and interactions between them. Furthermore, while current research mainly focuses on psychiatric symptoms and/or diagnoses as outcome variables, future research would benefit from broadening this scope towards variables estimating for example school, social and/or professional functioning in the COPMI’s (later) life. Second, protective factors that have been studied in the existing literature can be situated at three levels: the individual level (e.g., coping), the intrafamilial level (e.g., family functioning), and the contextual level (e.g., network support). In most included studies, however, the scope is limited to only one of these three levels. As a consequence, the results of these studies provide only fragmented and partial evidence for the processes underlying the adaptation of COPMI. Moreover, this fragmented approach is conceptually not in line with leading family stress models (see Weber, 2011), in which protective factors on all three levels are considered to be crucial to understand the varying effects of stressors (i.e., parental mental illness) on families and family members. Third, clear definitions of the constructs and variables under study are often missing. As a consequence, there is a lack of clarity in the differences and similarities between the concepts used in the reviewed studies (for example in the case of parenting concepts: parental warmth, parental sensitivity, parental acceptance and positive parenting were used interchangeably). The use of a theoretical framework, which was lacking in many of the included studies, may encourage clear distinction between the constructs under study. Fourth, in some of the included studies, the sample consisted of both COPMI and control children whose parents have no history of mental illness. Notwithstanding the value and importance of comparative studies, their methodological drawbacks need to be taken into account as well. In addition, in order to unravel the underlying factors and mechanisms that are protective in COPMI, it is equally important to examine effects *within* this COPMI group, and not only between groups. Focussing on a (sufficiently large) sample of COPMI may contribute to this field of inquiry in several ways. First, it can help to delineate competent functioning for this specific group of children. Competent functioning in the context of parental mental illness might be different from competent functioning in control families. Second, it might increase insight into the variability within this so-called “high-risk group”. Results of several included studies indicated that lower levels of a protective factor were reported by parents with a mental illness compared to control parents [e.g., Parenting style in van Loon et al., 2014; Family structure and support in Iacono et al., 2018; Coping in Thompson et al., 2017]. Based on these results, it would be premature to conclude that these factors are not protective in COPMI. Further research is needed to examine whether, within this COPMI group, the presence of these factors might protect children for worse outcome.

### Suggestions for future research

Future work should ideally rely on theoretical frameworks that simultaneously incorporate children’s individual strengths and vulnerabilities (individual level), family and parental factors (intrafamilial level) as well as the value of a supporting social network (contextual level) in order to best understand and predict child resilience in the context of a parental mental illness. Furthermore, both the variability within COPMI samples and between COPMI and control samples should be the focus of research, to gain best insight into those factors enhancing resilience. Finally, more homogenous samples or samples large enough to examine heterogeneity (e.g., age children, diagnosis parent) are recommended. Mixed qualitative and quantitative methods, along with observational methods and daily life studies (e.g., Ecological Momentary Assessment), are needed to assess the full range of relevant protective factors and outcomes.

### Implications for clinical practice

Based on this review, we can conclude that specific factors (i.e., information, support, family functioning and connectedness, child coping and parenting) play a role in helping children to better adapt to the mental illness of their parent. Protective factors to enhance resilience in COPMI are situated at the individual, the intrafamilial and the contextual level. Hence, health professionals and other persons involved should be aware that interventions can/should be directed to the child, the parent and the family as a whole.

In the following section, we will outline some potential “resilience builders” for each of the three levels. Some of which are comparable with the active elements of systemic interventions for child and adult focused problems (for more information, see Carr, 2019a, 2019b) or are already part of effective, evidence informed interventions implemented in clinical practice [e.g., The Family Talk Intervention; (Furlong et al., 2021)]. Before we describe these resilience builders, two points of attention for (clinical) practice are highlighted. (1) The first need is to identify these children. As difficulties in COMPI may seem secondary to the more pressing needs of the (mental illness of the) parent, such issues may be overlooked by psychosocial healthcare providers or may be seen as outside their purview of care. For each patient, the psychosocial healthcare providers should be aware of children living with that patient. They should inform about the wellbeing of the children as well as about the impact of the illness and treatment on the (lives of) the children (Reupert et al., 2022). Moreover, policy makers should consider ways to encourage such a registration and initial assessment of COPMI [e.g., through legislation, (Reedtz et al., 2022)]. (2) COPMI may already benefit when only one potential resilience builder is targeted. Indeed, even small actions by caregivers or other important adults in the child’s life can make a significant difference for these children.

Potential “resilience builders” for COPMI:

*Individual level*: information should be provided to COPMI and there are several ways in which this can be achieved. Empowering parents with a mental illness to give information to their own children, can be an impactful and meaningful way to both provide information to the children as well as to enhance connectedness between parent and child. If this is not (yet) possible, a professional can inform the children about the mental illness and the treatment (Beardslee et al., 1993). A low-threshold conversation tailored to the age and competences of the child is then recommended. In addition, for some children, teaching adaptive coping styles may be relevant. Children can be given simple tips and descriptions of how they can cope with specific situations they typically encounter, for instance through intervention programmes (Compas et al., 2010).

*Intrafamilial level*: results of the present review indicated that connectedness with parents as well as good parenting may foster resilience in children. Psychosocial healthcare providers can support parents (both the ill parent and the co-parent, if present) by including these themes (family functioning and parenting) in their consults (Beardslee, 2019). During these consults, whether/which tools the parents need to best inform and support the children can be addressed. In addition, improving communication about the mental illness within the family enhances connectedness and support within the family-system, and can thereby foster resilience in children (Beardslee, 2019). Also interventions aimed at strengthening the family functioning or enhancing positive family activities are valuable in this context (e.g., Compas et al., 2009). Finally, as concluded in this review, siblings can be very important to one another in the context of parental mental illness (Maybery et al., 2005; Bartsch et al., 2014; Kadish, 2015). It may be helpful to evaluate and talk about different ways in which siblings can support each other.

*Contextual level*: as social support from different sources (e.g., grandparents, friends, teachers) emerged as one of the most important protective factors, it is important to map the social network of the children and the family. This can be done together with the parent and the children. Ways in which the broader network can offer help to the family and the children can be discussed. Furthermore, a confidant to whom children can go to when their parent is unwell and with whom they can share their experiences can be an important source of support. Finally, getting in touch with other children who have shared life experiences can offer a unique kind of connection and support (Griffiths et al., 2012).

### Conclusion

Whereas a considerable amount of research has focused on the vulnerabilities in and risks for children growing up with a parent suffering from a mental illness, nowadays a growing body of research is exploring the factors that help build resilience in these children. In the present study, empirical studies on such protective factors were systematically reviewed and results were summarized. Five protective factors emerged from the available literature: Information, Support, Family functioning and Connectedness, Child coping, and Parenting. To further our understanding of the building blocks and underlying mechanisms of resilience in COPMI, additional rigorously designed studies are needed. Results from these empirical studies are essential in developing and optimizing programs of care for COPMI.

## Data availability statement

The original contributions presented in the study are included in the article/Supplementary Material, further inquiries can be directed to the corresponding author.

## Author contributions

MS and EL searched the databases and screened the selected articles. MS wrote a first draft of the manuscript. EL wrote parts of the manuscript. KS, LV, and GL provided meaningful feedback. All authors contributed to the article and approved the submitted version.
