# Supplementary material for: Protective factors enhancing resilience in children of parents with a mental illness: a systematic review
Source: Front Psychol. 2023 Dec 15;14:1243784. doi: 10.3389/fpsyg.2023.1243784 (PMC10773682; doi:10.3389/fpsyg.2023.1243784)
Supplement: Supplementary file 1 [file Table_1.pdf]

Supplementary Table 1.1 Overview of the ratings for scientific merit following the Mixed Method Appraisal Tool: Qualitative studies

Authors	S1 Are there clear research questions?	S2 Do the collected data allow to address the research questions?	1.1 Is the qualitative approach appropriate to answer the research question?	1.2 Are the qualitative data collection methods adequate to address the research question?	1.3 Are the findings adequately derived from the data?	1.4 Is the interpretation of results sufficiently substantiated by data?	1.5 Is there coherence between qualitative data sources, collection, analysis and interpretation?
**Bartsch *et al.*, 2014**	1	1	1	0	1	1	1
**Dunn, 1993**	1	1	1	1	1	1	1
**Griffiths *et al.*, 2012**	1	1	1	1	1	1	1
**Kadish, 2015**	1	1	1	1	1	1	1

Supplementary Table 1.2 Overview of the ratings for scientific merit following the Mixed Method Appraisal Tool: Quantitative randomized controlled trials

**Authors**	**S1 Are there clear research questions?**	**S2 Do the collected data allow to address the research questions?**	**2.1 Is randomization appropriately performed?**	**2.2 Are the groups comparable at baseline?**	**2.3 Are there complete outcome data?**	**2.4 Are outcome assessors blinded to the intervention provided?**	**2.5 Did the participants adhere to the assigned intervention?**
**Compas *et al*., 2010**	1	1	88	1	0	1	88

Supplementary Table 1.3 Overview of the ratings for scientific merit following the Mixed Method Appraisal Tool: Quantitative non-randomized studies

Authors	S1 Are there clear research questions?	S2 Do the collected data allow to address the research questions?	3.1. Are the participants representative of the target population	3.2 Are measurements appropriate regarding both the outcome and intervention (or exposure)?	3.3 Are there complete outcome data?	3.4 Are the confounders accounted for in the design and analysis	3.5 During the study period, is the intervention administered (or exposure occurred) as intended?)
**Black *et al.*, 2003**	1	1	1	1	1	0	88
**Charrois *et al.*, 2017**	1	1	88	1	88	1	1
**Feng *et al.*, 2008**	1	1	1	1	1	1	1
**Garber and Little, 1999**	1	1	1	1	1	1	1
**Iacono *et al.*, 2018**	1	1	1	1	0	1	88
**Jaser *et al.*, 2008**	1	1	1	1	1	1	1
**Jaser *et al.*, 2011**	1	1	1	1	88	0	1
**Lewandowski *et al.*, 2014**	1	1	1	1	1	1	0
**Loechner *et al.*, 2020**	1	1	1	1	0	1	1
**Monti and Rudolph, 2017**	1	1	1	1	1	1	0
**Riley *et al.*, 2009**	1	1	1	1	88	1	1
**Vakrat *et al.*, 2018**	1	1	1	1	1	1	1
**Van Loon *et al.*, 2014**	1	1	1	1	1	1	1
**Van Loon *et al.*, 2015**	1	1	1	1	1	1	88

Supplementary Table 1.4 Overview of the ratings for scientific merit following the Mixed Method Appraisal Tool: Quantitative descriptive studies

Authors	S1 Are there clear research questions?	S2 Do the collected data allow to address the research questions?	4.1 Is the sampling strategy relevant to address the research question?	4.2 Is the sample representative of teh target population?	4.3 Are the measurements appropriate?	4.4 Is the risk of nonresponse bias low?	4.5 Is the statistical analysis approproate to answer the research question?
**Boyd and Waanders, 2013**	1	1	1	1	1	1	1
**Chen, 2013**	1	1	1	1	1	1	0
**Collishaw *et al.*, 2016**	1	1	1	1	1	1	1
**Fear *et al.*, 2009**	1	1	1	1	1	88	1
**Foster *et al.*, 2008**	1	1	1	1	1	0	1
**Freed *et al.*, 2015**	1	1	1	1	1	88	1
**Garai *et al.*, 2009**	1	1	1	1	1	1	1
**Gruhn *et al.*, 2019**	1	1	1	1	1	1	1
**Havinga *et al.*, 2017**	1	1	1	1	1	88	1
**Jaser *et al.*, 2007**	1	1	1	1	1	1	1
**Keeton *et al.*, 2015**	1	1	1	88	1	1	1
**Langrock *et al.*, 2002**	1	1	1	1	1	1	1
**Mahedy *et al.*, 2018**	1	1	1	1	1	1	1
**Maybery *et al.*, 2005**	1	1	88	0	1	1	1
**Radicke *et al.*, 2021**	1	1	1	1	1	1	1

Authors	S1 Are there clear research questions?	S2 Do the collected data allow to address the research questions?	4.1 Is the sampling strategy relevant to address the research question?	4.2 Is the sample representative of teh target population?	4.3 Are the measurements appropriate?	4.4 Is the risk of nonresponse bias low?	4.5 Is the statistical analysis approproate to answer the research question?
**Schiffman *et al.*, 2002**	1	1	1	88	1	0	1
**Sellers *et al.*, 2014**	1	1	88	1	1	1	1
**Thompson *et al.*, 2017**	1	1	1	1	1	1	1
