# Supplementary material for: Protective factors enhancing resilience in children of parents with a mental illness: a systematic review
Source: Front Psychol. 2023 Dec 15;14:1243784. doi: 10.3389/fpsyg.2023.1243784 (PMC10773682; doi:10.3389/fpsyg.2023.1243784)
Supplement: Supplementary file 2 [file Table_2.PDF]

Supplemental Table 2 Summary of design characteristics and findings of all reviewed studies

Authors	Country of origin	Design	Sample Size[1]: Children, Parents, Clinicians, Teachers (female/male)	Parental mental illness	Protective factor(s)	Findings[2]
Bartsch *et al.*, 2014	Australia	Cross-sectional, Qualitative	*N* = 64 clinicians (52/4)	Borderline Personality Disorder (BPD)	Support, Information, Friends, Positive parenting	• The most commonly reported protective factors, as indicated by approximately 77% of respondents, was having a supportive and consistent role model and healthy social supports. Examples of supportive relationships included having another parent who did not have a diagnosis of BPD or other personality disorder, supportive siblings, a consistent extended family member (i.e. grandparent, aunt, etc.) or another stable role model in the community (i.e. school teacher, coach, youth worker, police officer, etc).<br>• A therapeutic intervention, ea. psycho-education about BPD and its symptoms, and insight in the parent' illness were indicated as helpful.<br>• Some child or parent characteristics were seen as potentially protective, such as having good social skills, participating in hobbies/activities (child characteristics) and validating their children's responses (parent characteristic)
Black *et al.*, 2003	USA	Longitudinal, Quantitative	*N* = 43 children (28/15), 21	Obsessive compulsive	Family Connectedne	• Children without disruptions in affective responsiveness within the family were

Authors	Country of origin	Design	Sample Size[1]: Children, Parents, Clinicians, Teachers (female/male)	Parental mental illness	Protective factor(s)	Findings[2]
			parents (14/7)	disorder	ss	less likely to have a diagnosis of broadly defined OCD (i.e., diagnosis of clinical as well as subclinical OCD).<br>• Other family functioning factors (i.e., family problem solving, family communication, family roles, affective involvement within the family) were not significant.
Boyd and Waanders, 2013	USA	Cross-sectional, Quantitative	*N* = 77 children (45/32), 77 parents (77/0)	Depression	Positive parenting, Friends, s-Support	• Positive parenting, child social skills (child and parent report) and support from extended family members (child report) were negatively and significantly associated with child depressive symptoms (correlations).<br>• Higher parent-reported child social skills were associated with lower depressive symptoms in children of parents with lower positive parenting skills (i.e. low positive parenting and low extent of parental involvement)<br>• When parents show low positive parenting skills (i.e., low positive parenting and low extent of parental involvement), high levels of parent-reported child social skills protect against child depressive symptoms.
Charrois *et al.*, 2017	Canada	Longitudinal, Quantitative	*N* = 265 parents (265/0)	Depression	Child-care quality	• Children of mothers with clinical depression after birth attending high-quality child-care presented fewer

**Authors**	**Country of origin**	**Design**	**Sample Size[1]: Children, Parents, Clinicians, Teachers (female/male)**	**Parental mental illness**	**Protective factor(s)**	**Findings[2]**
						difficulties (i.e., less hyperactivity and inattention) than those attending low-quality child-care.<br>• When attending high-quality child-care, hyperactivity and inattention scores did not differ between children whose mother was depressed vs. was never depressed.
Chen, 2013	USA	Longitudinal, Quantitative	*N* = 126 children (64/62), 126 parents (122/4)	Depression	Friends, Parental monitoring, Teacher support	• Prosocial friendships and parental monitoring were negatively associated with conduct disorder symptoms.<br>• Parental monitoring was negatively associated with youth school performance.<br>• Parental monitoring and teacher support were positively associated with youth educational aspirations.
Collishaw *et al.*, 2016	UK	Longitudinal, Quantitative	*N* = 331 children (194/115), 331 parents (309/22)	Depression	Parenting style, Support, Friends	• Parent positive expressed emotion, co-parent support, good-quality social relationships and frequent physical exercise (parent report) were associated with sustained good mental health.<br>• Co-parent support, good-quality social relationships (child and parent report) and out of school activities were related with mood resilience 4 years later.<br>• Parent warmth, parent positive

Authors	Country of origin	Design	Sample Size[1]: Children, Parents, Clinicians, Teachers (female/male)	Parental mental illness	Protective factor(s)	Findings[2]
						expressed emotion, co-parent support and good-quality social relationships (child and parent report) were related with behavioural resilience 4 years later.
Compas *et al.*, 2010	USA	Longitudinal, Quantitative	*N* = 111 children (47/64), 111 parents (95/16)	Depression	Coping, Positive parenting	• Significant effects for increases in secondary control coping, due to an intervention, were found for anxious-depressed symptoms (child report), internalizing symptoms (child report), and externalizing symptoms (parent report).<br>• Significant effects were found for increases in observed positive parenting, due to an intervention, on externalizing symptoms (parent report) and depressive symptoms (child report).
Dunn, 1993	USA	Cross-sectional, Qualitative	*N* = 9 children (5/4)	Psychosis, schizophrenia and/or bipolar disorder	Support	All participants identified one or more people whom they saw as supportive and helpful and who made a substantial difference in their lives (e.g. grandparents neighbors, family friends, teachers or coaches). These people were available on a somewhat regular basis and children saw them, and their home, as safe and welcoming.
Fear *et al.*, 2009	USA	Cross-sectional, Quantitative	N = 108 children (58/50), 108 parents	Depression	Coping	• The use of primary control coping was significantly related to less aggression (youth report only).

Authors	Country of origin	Design	Sample Size[1]: Children, Parents, Clinicians, Teachers (female/male)	Parental mental illness	Protective factor(s)	Findings[2]
			(91/17)			• The use of secondary control coping (acceptance, distraction, cognitive restructuring, positive thinking) was significantly related to both less anxiety/depression symptoms and less aggression.
Feng *et al.*, 2008	USA	Cross-sectional, Quantitative	*N* = 37 children (22/15), 33 parents (33/0)	Depression	Positive parenting	Among children of mothers with depression, maternal positivity was *not* related to higher positive mood (joy) and active emotion regulation.
Foster *et al.*, 2008	USA	Longitudinal, Quantitative	*N* = 114 children (61/53), 114 parents (114/0)	Depression	Positive parenting, Family functioning	• Maternal acceptance measured at 3-month follow-up was negatively related to youth internalizing symptoms measured at the same time point. Moreover maternal acceptance mediated the relationship between maternal remission and youth internalizing symtoms.<br>• At the 3-month follow-up, maternal psychological control measured was positively related to youth internalizing symptoms.<br>• Higher cohesion and expressiveness, and lower conflict (i.e., family functioning) were significantly related to improvements in youth internalizing problems.<br>• There was no significant relationship between maternal acceptance, maternal

Authors	Country of origin	Design	Sample Size[1]: Children, Parents, Clinicians, Teachers (female/male)	Parental mental illness	Protective factor(s)	Findings[2]
						psychological control, family functioning, and externalizing problems.
Freed *et al.*, 2015	USA	Cross-sectional, Quantitative	*N* = 117 children (55/62), 75 parents (51/24)	Bipolar disorder	Family connectednes s	• Higher family cohesion was associated with lower internalizing and externalizing symptoms in offspring (correlation)<br>• In younger offspring (under 13 years old), higher levels of cohesion predicted lower internalizing symptoms. In older offspring family cohesion was not significantly associated with internalizing symptoms. (regression analyses).<br>• Offspring without mood disorders had higher mean scores for family cohesion.<br>• No association between family expressiveness and any outcome measures.
Garai *et al.*, 2009	USA	Cross-sectional, Quantitative	*N* = 84 children (41/44), 65 parents (65/0)	Depression	Positive parenting	• Maternal sensitivity (observed during mother-child interaction in stressful context) was significantly associated with child report of externalizing symptoms, such that higher levels of maternal sensitivity was related to lower levels of externalizing symptoms.<br>• Maternal sensitivity was not significantly associated with child reported internalizing symptoms. However, high levels of maternal

Authors	Country of origin	Design	Sample Size[1]: Children, Parents, Clinicians, Teachers (female/male)	Parental mental illness	Protective factor(s)	Findings[2]
						depressive symptoms **OR** low levels of maternal sensitivity led to similar levels of internalizing symptoms. The findings suggest that maternal insensitivity may be conceptualized as a stressor similar to maternal depressive symptoms.
Garber and Little, 1999	USA	Longitudinal, Quantitative	*N* = 51 children (37/14), 51 parents (51/0)	Depression	Coping, Family functioning, Support	• High-competence children (i.e. high functioning and without psychopathology) used more positive coping when compared with the decreased-competence children.<br>• High-competence children reported significantly better family relationships and marginally significantly higher maternal acceptance than did the decreased-competence children.<br>• High-competence group reported higher levels of support from family members and close friends than did the decreased-competence children<br>• Children who had experienced a high level of school hassels were more likely to be in the high-competence group if they had better family relationships (moderator).
Griffiths *et al.*, 2012	UK	Cross-sectional, Qualitative	*N* = 10 children (5/5)	OCD	Information, Support	• Several children felt that they should be told about their parent's difficulties. They were annoyed by the lack of recognition of the difficulties they faced

Authors	Country of origin	Design	Sample Size[1]: Children, Parents, Clinicians, Teachers (female/male)	Parental mental illness	Protective factor(s)	Findings[2]
						and felt more information and support would have helped.<br>• Family members seemed very important, particularly for performing everyday tasks left by the parent with OCD.<br>• Outside the family, the children were selective and felt that most people did not understand and could not help. To manage this, some spoke to people with ‘expertise’; either a professional or a friend who knew more through shared experience.
Gruhn *et al.*, 2019	USA	Cross-sectional, Quantitative	*N* = 160 children (80/80), 160 parents (160/0)	Depression	Coping	• During mother-child interactions about a stressful event, secondary control coping was positively related to positive mood and negatively to observed hostility (both significant), when controlling for other types of coping, mothers’ depressive symptoms, and child gender. Secondary control coping was not related to anxiety or sadness.<br>• The positive and negative associations between secondary control coping and respectively positive mood and hostility respectively remained significant after controlling for observed emotions during mother-child interactions about a pleasant event.<br>• The association of secondary control

Authors	Country of origin	Design	Sample Size[1]: Children, Parents, Clinicians, Teachers (female/male)	Parental mental illness	Protective factor(s)	Findings[2]
						coping with positive mood expression remained significant within separate regression models for families of mothers with current depression and past depression.
Havinga *et al.*, 2017	The Netherlands	Longitudinal, Quantitative	*N* = 523 children	Depression and/or anxiety	Family functioning	• A balanced family functioning (i.e. separated/connected family cohesion and structured/flexible family adaptation) was associated with a significantly decreased hazard of offspring risk for onset of mood and/or anxiety disorder.
Iacono *et al.*, 2018	Canada	Longitudinal, Quantitative	*N* = 145 children[3] (76/69), 103 parents (?/?)	Bipolar disorder	Support, Structure, Control	• Higher levels of structure in middle childhood was associated with lower rates of parent-reported internalizing and externalizing symptom, teacher-reported externalizing symptoms and clinician-rated clinical symptom.<br>• High levels of control in middle childhood predicted lower rates of offspring substance use symptoms in late adolescence and young adulthood.<br>• Conversely, high levels of structure in middle childhood predicted *higher* rates of depressive symptoms in late adolescence and young adulthood.<br>Mediation analyses were also conducted:<br>• Structure negatively mediated the association between offspring risk status

Authors	Country of origin	Design	Sample Size[1]: Children, Parents, Clinicians, Teachers (female/male)	Parental mental illness	Protective factor(s)	Findings[2]
						and rates of internalizing symptoms, externalizing symptoms (parent- and teacher reported) and clinician rated clinical symptoms (cross-sectional).<br>• Support and control negatively significantly mediated the association between offspring risk status and parent-reported rates of externalizing symptoms (cross-sectional).<br>• Control in middle childhood negatively mediated the association between offspring risk status and the number of symptoms of depressive and substance use disorder 12 years later.<br>• Structure in middle childhood positively mediated the association between offspring risk status and the number of symptoms of depressive disorder 12 years later.
Jaser *et al.*, 2007	USA	Cross-sectional, Quantitative	*N* = 73 children (35/38), 50 parents (46/4)	Depression	Coping	• Greater use of secondary control coping with family stress and peer stress predicted fewer child reported symptoms of anxiety and depression.<br>• While greater use of primary control coping with peer stress predicted fewer symptoms (anxiety/depression and aggression), greater use of primary control coping with family stress predicted more child reported symptoms

Authors	Country of origin	Design	Sample Size[1]: Children, Parents, Clinicians, Teachers (female/male)	Parental mental illness	Protective factor(s)	Findings[2]
						of anxiety and depression.<br>• Adolescent coping did not significantly predict symptoms (anxiety/depression and aggression) as reported by the parent(s).
Jaser *et al.*, 2008	USA	Cross-sectional, Quantitative	*N* = 72 children (36/36), 72 parents (72/0)	Depression	Coping	• Greater use of secondary control coping was significantly related to fewer depressive symptoms and fewer symptoms of affective problems and oppositional defiant problems in adolescents.<br>• Secondary control coping mediated the association between observed maternal sadness and child symptoms (depressive symptoms, affective problems and oppositional defiant problems).
Jaser *et al.*, 2011	USA	Cross-sectional, Quantitative	*N* = 72 children (?/?), 72 parents (72/0)	Depression	Coping	• Greater use of primary control coping was associated with higher levels of observed positive mood and lower levels of affective problems. (correlation)<br>• Greater use of secondary control coping was associated with higher levels of observed positive mood and lower levels of affective problems in adolescents. (correlation)<br>• However, coping was not significantly related to observed sadness in adolescents. (correlation)<br>• Higher levels of observed positive mood

Authors	Country of origin	Design	Sample Size[1]: Children, Parents, Clinicians, Teachers (female/male)	Parental mental illness	Protective factor(s)	Findings[2]
						predicted lower levels of affective problems, after accounting for maternal history of depression and current maternal depressive symptoms.
Kadish, 2015	South Africa	Cross-sectional, Qualitative	*N* = 5 children (5/0)	Psychosis	Support, Coping	• Father = a stable, trustworthy parent<br>• Siblings were often perceived to be a source of support as well as sounding boards to help each other through troubled times.<br>• Live- in domestic worker played a fundamental role in physical and psychological survival and provided consistent and nurturing care.<br>• Children believed they possessed the necessary resilience to survive their childhood because they used different coping behaviours (intelligence, caring for themselves and others and an active coping style).
Keeton *et al.*, 2015	USA	Cross-sectional, Quantitative	*N* = 81 children (47/34), 81 parents (62/19)	Anxiety	Connectedness with siblings	Parent psychological distress was not associated with child psychological symptoms in children reporting a good quality sibling relationship (i.e., high companionship, low conflict), whereas a significant positive association between parent psychological distress and child psychological symptoms was found in children reporting a poor quality sibling relationship. Thus, good quality sibling

Authors	Country of origin	Design	Sample Size[1]: Children, Parents, Clinicians, Teachers (female/male)	Parental mental illness	Protective factor(s)	Findings[2]
						relationships protect against the negative outcomes associated with parental psychological distress.
Langrock *et al.*, 2002	USA	Cross-sectional, Quantitative	*N* = 101 children (51/50), 66 parents (56/10)	Depression	Coping	Based on parent report:<br>• Secondary control coping predicted decreased child anxiety/depressive symptoms, but not aggression symptoms.<br>• Primary control coping did not significantly predict these child symtoms (anxiety/depression, agression).<br>• To note: Secondary control coping mediated the relationship between parental withdrawal and intrusiveness, and child symptoms. Thus, although secondary control coping is associated with less anxiety/depression in children, children confronted with higher levels of parental withdrawal (i.e. stress associated with parental depression) are less likely to use this form of adaptive coping to manage stressors.
Lewandowski *et al.*, 2014	USA	Longitudinal, Quantitative	*N* = 115 children (?/?), 50 families with depression (66 depressed	Depression	Parenting, Family functioning	• Resilience, defined by consistent high functioning, was significantly associated with maternal overprotection: Offspring who had less overprotective mothers at baseline had significantly greater odds

**Authors**	**Country of origin**	**Design**	**Sample Size[1]: Children, Parents, Clinicians, Teachers (female/male)**	**Parental mental illness**	**Protective factor(s)**	**Findings[2]**
			mothers, 27 depressed fathers, 22 depressed mother and father)			of resilient outcome.<br>• Family cohesion and maternal affection did not significantly predict resilience (defined by consistent high functioning).<br>• Resilience, defined by absence of lifetime psychiatric diagnosis was not significantly associated with maternal affection, maternal overprotection or family cohesion.
Loechner *et al.*, 2020	Germany	Cross-sectional, Quantitative	*N* = 111 children (63/48)	Depression	Positive parenting	• A less positive parenting style was perceived in the high risk group (children of parents with depression) compared to the low risk group (control).<br>• Perceived parenting style was not significantly related to children's depressive symptoms after accounting for parental depression.<br>• Perceived parenting style did not mediate the significant association between parental depression and child depressive symptoms
Mahedy *et al.*, 2018	UK	Longidtunal, Quantitative	*N* = 265 children (119/146), 265 parents (265/0)	Depression	Support, Couple connectednes s	• High paternal emotional support at baseline was associated with fewer symptoms of depression and reduced likelihood of adolescent psychiatric disorder at follow-up; but not with disruptive behaviours at follow-up.<br>• Adolescent symptoms of depression at

Authors	Country of origin	Design	Sample Size[1]: Children, Parents, Clinicians, Teachers (female/male)	Parental mental illness	Protective factor(s)	Findings[2]
						baseline did not predict paternal emotional support at follow up, ruling out that protective effects were due to reverse causation.<br>• Path-analysis showed evidence of an indirect pathway whereby couple relationship quality negatively predicted paternal emotional support which in turn negatively predicted adolescent symptoms of depression, but not disruptive behaviours.
Maybery *et al.*, 2005	Australia	Cross-sectional, Mixed	*N* = 12 children (?/?), 10 parents (?/?) and 62 mental health professionals (?/?)	Mood disorder, anxiety disorder, personality disorder and psychotic disorder	Information, Support	• The majority of parents saw siblings as being a great support to one another and particularly as someone to rely on when the parent is unwell. In addition, several children said they felt their brother and/or sister were very supportive and that they could rely on them when things were not going well.<br>• Majority of parent participants commented on the need for more support for the children, particularly from professionals<br>• While their children knew something about mental illness, they could benefit from more education.<br>• Friendships were of great importance to the child participants. Most of the children said that their relationships with

Authors	Country of origin	Design	Sample Size[1]: Children, Parents, Clinicians, Teachers (female/male)	Parental mental illness	Protective factor(s)	Findings[2]
						friends were positive and supportive. Some children also said that they went to a friend's house as a way of coping when their parent is unwell.
Monti and Rudolph, 2017	USA	Longitudinal, Quantitative	*N* = 165 children (85/80), 165 parents (165/0)	Depression	Coping	• Adaptive responses to interpersonal stressors with peers (high effortful engagement = primary and secondary control coping) buffers the effect of maternal depression on initial levels (for girls) and trajectories (for boys) of youth depression. In other words:<br>• For girls, high effortful engagement mitigates the negative effect of maternal depression on youth depression, both at baseline and follow-up<br>• For boys, high effortful engagement buffers the negative effect of maternal depression on trajectories of youth depression (i.e., when exposed to maternal depression, the use of high effortful engagement led to decreases in youth depression over time)
Radicke *et al.*, 2021	Germany and Switzerland	Cross-sectional, Quantitative	*N* = 198 children (110/88), 134 parents (102/32)	ICD-10 psychiatric diagnoses	Support, Family functioning	• Social support was associated with higher health-related quality of life in children and adolescents<br>• There was no significant association

Authors	Country of origin	Design	Sample Size[1]: Children, Parents, Clinicians, Teachers (female/male)	Parental mental illness	Protective factor(s)	Findings[2]
						between family functioning and health-related quality of life
Riley *et al.*, 2009	USA	Cross-sectional, Quantitative	*N* = 133 (70/63), 133 parents (133/0), 83 co-parents/father figure (0/83), 89 teachers)	Depression (71% had comorbid anxiety disorders)	Positive parenting, Parenting quality, Family functioning	• More positive parenting was associated with significantly fewer child emotional and behavioural problems and more child adaptive skills.<br>• Parenting quality appeared to mediate partially the association between maternal depression and children's emotional and behavioural problems, as reported by both mothers and fathers.<br>• Parenting quality fully mediated the association between maternal depression and child adaptive skills.<br>• Family environment was not a significant predictor of children's emotional and behavioural problems or adaptive skills, when maternal depression was controlled in the models.
Schiffman *et al.*, 2002	USA and Denmark	Longitudinal, Quantitative	*N* = 110 children (?/?)	Schizophrenia	Connectedness with parents	Good relationships with both parents are associated with decreased rates of schizophrenia in adult offspring: among offspring with poor parental relations, 40.7% received an adult diagnosis of schizophrenia; among offspring with good parental relations, only 9.8%.
Sellers *et al.*, 2014	UK	Longitudinal, Quantitative	*N* = 299 (174/125), 299	Depression	Parenting	• Maternal warmth (T2, 16 months later) was significantly negatively associated

Authors	Country of origin	Design	Sample Size[1]: Children, Parents, Clinicians, Teachers (female/male)	Parental mental illness	Protective factor(s)	Findings[2]
			parents (299/0)			with child disruptive symptoms at T3/28 months later, but not with child depressive symptoms.<br>• Moreover, maternal warmth mediated the relationship between maternal depression severity and child disruptive symptoms.
Thompson *et al.*, 2017	USA	Cross-sectional, Quantitative	*N* = 122 children[3] (122/0), 122 parents (122/0)	Depression	Coping	Higher use of adaptive coping (i.e. primary and secondary control coping; mothers' and daugthers report) was significantly associated with less daughers' depressive symptoms.<br>Mediation analyses were also conducted:<br>• Daughters' self-reported adaptive coping mediated the association between mothers' depressive symptoms and daughters' depressive symptoms.<br>• Daughters' adaptive coping, as reported by the mothers, mediated the association between mothers' depressive symptoms and daughters' depressive symptoms.<br>• To note: the mediating effect of adaptive coping on the relation between mothers' depressive symptoms and daughters' self-reported depressive symptoms was only present for daughters who were at a later stage of pubertal maturation (moderated mediation).

Authors	Country of origin	Design	Sample Size[1]: Children, Parents, Clinicians, Teachers (female/male)	Parental mental illness	Protective factor(s)	Findings[2]
Vakrat *et al.*, 2018	Israel	Cross-sectional, Quantitative	*N* = 149 children (?/?), 149 parents (149 /0), 149 co-parents (0/149)	Depression	Support	Among children of mothers with depression, children experiencing sensitive fathering (e.g., acknowledging, social gaze, vocalization, positive affect, supportive presence, resourcefulness, praising) were significantly less likely to receive a clinical diagnosis upon school entry as compared to children of depressed mothers whose fathers were less sensitive.
Van Loon *et al.*, 2014	The Netherlands	Cross-sectional, Quantitative	*N* = 251 children[3] (123/128), 251 parents (188/63)	'Mental illness' (e.g., mood problem, anxiety problem, personality disorder, etc.)	Parenting, Family functioning	• More parental monitoring was associated with less adolescent's externalizing problems<br>• No significant association was found between parental support, family cohesion, family expressiveness on the one hand and adolescent's outcomes (internalizing and externalizing problems) on the other hand<br>Path analyses were also conducted:<br>• Having a parent with a mental illness was directly related to having more internalizing problems in adolescence, but no longer to more externalizing problems after inclusion of the family factors.<br>• Families with a mentally ill parent showed less monitoring of the child and more family conflict, which in turn, was associated with adolescents'

Authors	Country of origin	Design	Sample Size[1]: Children, Parents, Clinicians, Teachers (female/male)	Parental mental illness	Protective factor(s)	Findings[2]
						externalizing problems.
Van Loon *et al.*, 2015	The Netherlands	Longitudinal, Quantitative	*N* = 234 children (113/121), 234 parents (177/57)	'Mental illness' (e.g., mood problem, anxiety problem, personality disorder, etc.)	Coping, Support, Parenting, Family functioning	• High parental monitoring was related to fewer externalizing problems at baseline and predicted fewer internalizing problems 2 years later.<br>• Active coping strategy, parental support, and the broader family functioning (i.e., family cohesion, family expressiveness, family conflict, and perceived family support) were not related to internalizing or externalizing problems, nor predicted internalizing problems over time.<br>• The use of confrontation as an active coping style predicted fewer internalizing problems 2 years later.<br>• Active coping strategy, parental monitoring, parental support, and the broader family functioning (i.e., family cohesion, family expressiveness, family conflict, and perceived family support) did not predict externalizing problems over time.

Notes: [1] 'Parent' refers to the parent with a mental illness diagnosis, 'Co-parent' refers to the healthy parent; [2]Only findings relevant to this systematic review are reported; [3]Sample consisted of both children of a parent with a mental illness and control parents. Parental mental illness is considered as independent variable in the mediation analysis. However, in the regression analyses, no distinction between both groups were made.
