# Supplementary figures and images for: Protective factors enhancing resilience in children of parents with a mental illness: a systematic review
Source: Front Psychol. 2023 Dec 15;14:1243784. doi: 10.3389/fpsyg.2023.1243784 (PMC10773682; doi:10.3389/fpsyg.2023.1243784)

**Fig. 1** Flow chart

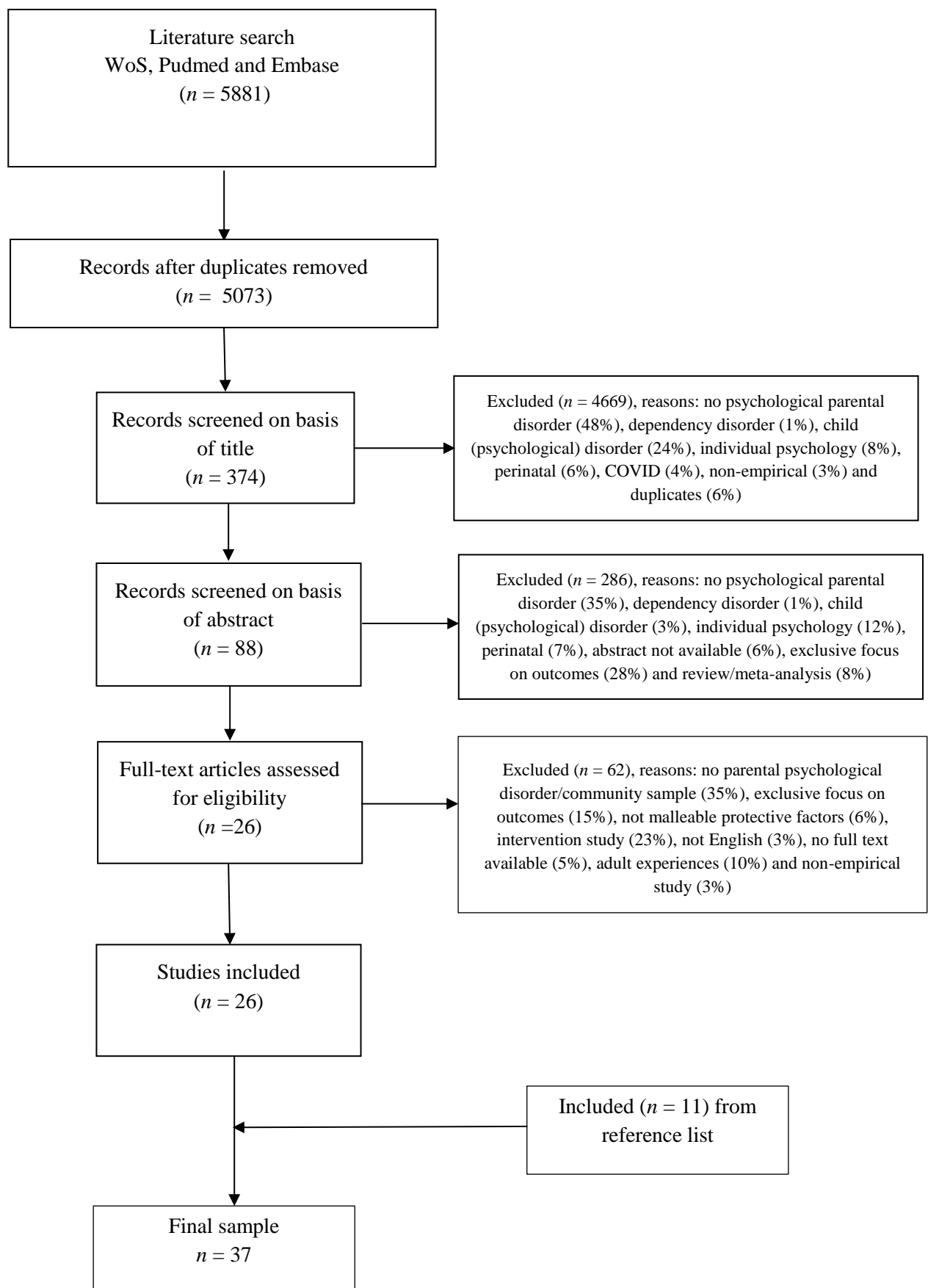

Supplement: Supplementary file 3 [file Image_1.PDF]
